# MRI background parenchymal enhancement, fibroglandular tissue, and mammographic breast density in patients with invasive lobular breast cancer on adjuvant endocrine hormonal treatment: associations with survival

**DOI:** 10.1186/s13058-020-01329-z

**Published:** 2020-08-20

**Authors:** Roberto Lo Gullo, Isaac Daimiel, Carolina Rossi Saccarelli, Almir Bitencourt, Varadan Sevilimedu, Danny F. Martinez, Maxine S. Jochelson, Elizabeth A. Morris, Jeffrey S. Reiner, Katja Pinker

**Affiliations:** 1 Department of Radiology, Breast Imaging Service, Memorial Sloan Kettering Cancer Center, 300 E 66th Street, New York, NY 10065 USA; 2 Department of Epidemiology and Biostatistics, Memorial Sloan Kettering Cancer Center, 485 Lexington Avenue, New York, NY 10017 USA; 3 Department of Biomedical Imaging and Image-guided Therapy, Molecular and Gender Imaging Service, Medical University of Vienna, Waehringer Guertel 18-20, 1090 Wien, Austria

**Keywords:** Breast cancer, Imaging, Background parenchymal enhancement, Invasive lobular, Survival

## Abstract

**Background:**

To investigate if baseline and/or changes in contralateral background parenchymal enhancement (BPE) and fibroglandular tissue (FGT) measured on magnetic resonance imaging (MRI) and mammographic breast density (MD) can be used as imaging biomarkers for overall and recurrence-free survival in patients with invasive lobular carcinomas (ILCs) undergoing adjuvant endocrine treatment.

**Methods:**

Women who fulfilled the following inclusion criteria were included in this retrospective HIPAA-compliant IRB-approved study: unilateral ILC, pre-treatment breast MRI and/or mammography from 2000 to 2010, adjuvant endocrine treatment, follow-up MRI, and/or mammography 1–2 years after treatment onset. BPE, FGT, and mammographic MD of the contralateral breast were independently graded by four dedicated breast radiologists according to BI-RADS. Associations between the baseline levels and change in levels of BPE, FGT, and MD with overall survival and recurrence-free survival were assessed using Kaplan–Meier survival curves and Cox regression analysis.

**Results:**

Two hundred ninety-eight patients (average age = 54.1 years, range = 31–79) fulfilled the inclusion criteria. The average follow-up duration was 11.8 years (range = 2–19). Baseline and change in levels of BPE, FGT, and MD were not significantly associated with recurrence-free or overall survival. Recurrence-free and overall survival were affected by histological subtype (*p* < 0.0001), number of metastatic axillary lymph nodes (*p* < 0.0001), age (*p* = 0.01), and adjuvant endocrine treatment duration (*p* < 0.001).

**Conclusions:**

Qualitative evaluation of BPE, FGT, and mammographic MD changes cannot predict which patients are more likely to benefit from adjuvant endocrine treatment.

## Background

Invasive lobular carcinomas (ILCs) constitute approximately 10% of breast cancers [1] and have similar or worse outcomes compared with stage-matched invasive ductal carcinomas (IDC) [2, 3] even though they often present with good prognostic features (low-grade estrogen- and progesterone-positive tumors and negative HER2 protein amplification). They possess a different histologic and genetic makeup from IDCs, presenting with small non-cohesive cells that grow in a linear fashion and infiltrate the stroma. They lack E-cadherin protein expression [4] and commonly present with loss of PTEN, activation of AKT, and mutations in TBX3 and FOXA1 [5]. Data indicate that ILCs derive less benefit from conventional chemotherapy [6–10] but have superior benefit from hormonal therapy [11] compared with stage-matched IDCs.

ILCs are more commonly mammographically occult (14.8% vs 1.2%) and more likely present as mass lesions compared to IDCs (59.2% vs 44.7%) [12]. On magnetic resonance imaging (MRI), both ILCs and IDCs most commonly present as spiculated or irregular masses with heterogeneous enhancement. Multifocality is more frequently associated with ILCs (40.7%) than with IDCs (14.1%) [12]. The mandatory assessment of the amount of fibroglandular tissue (FGT) and background parenchymal enhancement (BPE) was included in the latest edition of the Breast Imaging Reporting and Data System (BI-RADS) MRI lexicon [13–15]. FGT is the equivalent to mammographic breast density (MD), and BPE is defined as the enhancement of fibroglandular tissue on breast MRI after intravenous administration of a contrast agent [16]. BPE, MD, and to a lesser extent FGT have been proposed as imaging biomarkers to predict the risk of breast cancer, likelihood of local recurrence, response to neoadjuvant chemotherapy, and survival [17–22]. Studies have demonstrated that FGT, MD, and BPE can be used interchangeably in certain settings [23, 24]. However, those studies have focused on only ductal breast cancers (invasive and in situ) or on both ductal and lobular as a single entity (with the latter being less commonly represented across the studies).

The purpose of our study was to investigate if baseline and/or changes in contralateral BPE and FGT on MRI and mammographic MD after adjuvant endocrine therapy can be used as imaging biomarkers for overall and recurrence-free survival in patients with ILC. As it is well known that evaluation of breast density, fibroglandular tissue, and BPE is subjective limiting the reproducibility of studies such as this, as a secondary aim, we assessed inter-reader agreement among 4 breast radiologists.

## Methods

### Patients

This retrospective, single-center study was Health Insurance Portability and Accountability Act-compliant and received Institutional Review Board approval.

Consecutive women who fulfilled the following inclusion criteria were included: biopsy-proven ILC including classic, pleomorphic, and mixed ductal, and lobular subtypes with predominant lobular features; pre-treatment contrast-enhanced MRI and/or mammography between 2000 and 2010; adjuvant hormonal treatment for a minimum of 6 months; and available MRI and/or mammography 1–2 years after the start date of endocrine treatment.

Exclusion criteria were patients with prior invasive breast cancer, bilateral cancers, prior contralateral breast surgery/radiotherapy or a follow-up of less than 4 years; patients with mixed ductal and lobular carcinomas with predominant ductal features; and patients with triple-negative tumors.

We recorded the tumor diameter that was measured on pathology, hormone receptor status, number of metastatic lymph nodes, type and duration of endocrine treatment, and concurrent chemotherapy and radiotherapy treatment. It was not possible to record the menopausal status for every patient as this was a retrospective study and this information was not always stated in the medical record.

### MRI examination

Dynamic contrast-enhanced MRI was performed using a 3-T or 1.5-T unit with a dedicated breast coil (Signa; General Electric, Waukesha, WI, USA) using a state-of-the-art MR imaging protocol which included fat-suppressed T2-weighted, non-fat-suppressed T1-weighted, and fat-suppressed T1-weighted sequences before and after intravenous administration of gadolinium-based contrast agent [25, 26]. Subtraction and 3D reconstruction images were generated.

### MRI interpretation

Four dedicated breast radiologists (RLG, ID, CS, and AB), each with 4 years of experience, independently reviewed fat-suppressed contrast-enhanced and non-contrast-enhanced T1-weighted sagittal images of the unaffected contralateral breast of each patient before and after adjuvant endocrine treatment to assess BPE and FGT of the baseline scan. BPE of the unaffected contralateral breast was classified according to the BI-RADS lexicon (Additional File 1, Fig. A1) as minimal, mild, moderate, or extreme. FGT of the unaffected contralateral breast was classified according to the BI-RADS lexicon as fatty (the breasts are almost entirely fatty), scattered (there are scattered areas of fibroglandular density), heterogeneously dense (the breasts are heterogeneously dense, which may obscure small masses), or dense (the breasts are extremely dense, which lowers the sensitivity of mammography). On the first post-treatment MRI after the onset of adjuvant endocrine treatment, change in both FGT and BPE was classified as minimal/no change or moderate/marked decrease (Additional File 2, Fig. A2; and Additional File 3, Fig. A3), with the latter being a change in category (i.e., from moderate to mild or minimal).

### Mammography examination and interpretation

The same four dedicated breast radiologists (RLG, ID, CS, and AB) reviewed the full-field digital mammography in the cranio-caudal and oblique view of the contralateral breast of each patient before and after endocrine treatment. MD was classified according to ACR guidelines as fatty, scattered, heterogeneous, and dense. On the first post-treatment mammogram after the onset of endocrine treatment, change in MD was classified as minimal/no change or moderate/marked decrease, with the latter being a change in category (i.e., from dense to heterogeneous or scattered or fatty).

### Statistical analysis

The primary aim of this study was to assess if a reduction in contralateral BPE, FGT, or MD is associated with overall and recurrence-free survival in patients with ILC receiving adjuvant endocrine treatment. The associations were assessed using Kaplan–Meier survival curves and Cox regression analysis. Recurrences were recorded as (1) “local” in cases of recurrence in the ipsilateral breast, (2) “regional” in cases of recurrence in the ipsilateral axilla and regional lymph nodes, (3) “local contralateral” in cases of recurrence of lobular cancer in the contralateral breast, (4) “contralateral primary of different histology” (i.e., ductal carcinoma in situ or invasive ductal carcinoma), or (5) “distant” in all other cases. The date and cause of death were also noted.

To determine the inter-reader agreement in both imaging findings and change in imaging findings, Kendall rank correlation coefficient was used. This coefficient was used because each reader’s imaging report as well as change in imaging report was based on a rank measure that increased with increasing severity of the lesion (ranging from 1 to 4) and increasing level of improvement from the previous report (ranging from 0 to 4), respectively. Kendall rank correlation coefficient was interpreted as follows: 0, no agreement; 0–0.20, slight agreement; 0.21–0.40, fair agreement; 0.41–0.60, moderate agreement; 0.61–0.80, substantial agreement; and 0.81–1.0, almost perfect agreement. All statistical analyses were conducted using SAS 9.4 (SAS Institute, Cary, NC, USA) and R 3.5.3 (R Core Development Team, Vienna, Austria).

In multivariable Cox regression analysis, patient age, endocrine treatment duration, type of endocrine treatment received, radiotherapy, chemotherapy, tumor diameter, number of metastatic lymph nodes involved, focality of the tumor, histology, and hormone receptor status were used as covariates, with recurrence-free survival and overall survival as the outcome variables. The final model was selected using a backward elimination technique where a covariate with a *p* value of 0.05 or lower would be eligible for exclusion from the model. In order to account for inter-rater correlation, we treated each reader’s rating as a fixed effect and calculated robust (sandwich) covariance estimates using PROC PHREG in SAS 9.4. Kaplan–Meier plots were also constructed using BPE and FGT categories as strata, to assess differences in patterns of overall and recurrence-free survival.

## Results

### Patient and lesion characteristics

Patient and lesion characteristics are summarized in Table 1. We included 298 patients (average age = 54.1 years, SD = 9.4, range = 31–79). The average follow-up duration was 11.8 years (SD = 3.2, range = 2–19). Of 298 patients, 229/298 had classic lobular histology, 31/298 had pleomorphic histology, and 38/298 had mixed ductal and lobular histology. All patients were treated with endocrine treatment: 139 patients were treated with an aromatase inhibitor (AI), 156 patients with a selective estrogen receptor modulator (SERMs), and 3 patients with a luteinizing hormone-releasing hormone (LHRH) agonist for an average duration of 6.8 ± 2.6 years (range 0.5–15 years). In addition to endocrine treatment, 193/298 patients underwent chemotherapy and 200/298 underwent radiotherapy. Two hundred forty-five patients were evaluated with MRI, 112 patients were evaluated with mammography, and 60 patients were evaluated with both mammography and MRI.

**Table 1:** Patient and lesion characteristics

Patients	Total (***n*** = 298)	First recurrence (***n*** = 50)	Second recurrence (***n*** = 6)	Death (***n*** = 21)*
Histology				
Lobular	229	38	3	14
Pleomorphic	31	8	2	6
Mixed	38	4	1	1
Focality				
Unifocal	122	17	1	10
Bifocal/multifocal	175	33	5	10
Unknown	1	0	0	1
Tumor diameter				
2–19 mm	212	35	3	8
20–49 mm	71	8	1	7
> 50 mm	15	7	2	6
Axillary load				
None	192	26	3	7
1–3	84	13	1	3
> 4	21	11	2	10
Unknown	1	0	0	1
Hormone receptor				
ER/PR+ HER2−	292	49	6	21
HER2+	6	1	0	0
Endocrine treatment				
AI	139	19	3	10
SERMS	156	31	3	11
LHRH analogues	3			
Endocrine treatment duration				
≤ 5 years	145	32	4	16
≥ 6 years	153	18	2	5
Concurrent chemotherapy and radiotherapy				
Chemotherapy	193	38	5	19
Radiotherapy	200	36	5	14
Chemo + radio	126	28	4	12
No chemo-/radiotherapy	31	4	0	0

*Abbreviations*: *AI* aromatase inhibitors, *SERMS* selective estrogen receptor modulators, *LIRH* luteinizing hormone-releasing hormone

*Eight patients’ death was not directly related to breast cancer

Table 2 shows the MRI and mammography findings for each reader assessing BPE, FGT, and MD.

**Table 2:** Results from the MRI and mammographic evaluation for each reader in terms of background parenchymal enhancement (BPE), fibroglandular tissue (FGT), and mammographic breast density (MD)

	BPE	FGT	MD
**Reader 1**	*n* = 245	*n* = 245	*n* = 112
A	119	16	4
B	66	42	15
C	48	133	79
D	12	54	14
**Reader 2**	*n* = 243	*n* = 243	*n* = 112
A	92	13	1
B	76	81	30
C	56	101	69
D	19	48	12
**Reader 3**	*n* = 245	*n* = 245	*n* = 112
A	45	5	0
B	116	56	23
C	71	138	74
D	13	46	15
**Reader 4**	*n* = 244	*n* = 244	*n* = 112
A	93	4	1
B	93	57	16
C	44	132	83
D	14	51	12

“*n*” denotes the number of observations. BPE: “A” minimal, “B” mild, “C” moderate, and “D” marked. FGT and MD: “A” fatty (< 25% of breast comprised glandular tissue), “B” scattered (25–50% of breast comprised glandular tissue), “C” heterogeneously dense (51–75% of breast comprised glandular tissue), and “D” dense (> 75% of breast comprised glandular tissue). Reader 2 and Reader 4 reported BPE and FGT of 243 and 244 patients, respectively, because they did not feel they could make a correct assessment in the change in BPE and FGT due to the presence of breast implants

On baseline MRI performed before the start of endocrine therapy, most patients presented with minimal/mild BPE: 185 patients (76%) for R1, 168 patients (69%) for R2, 161 patients (66%) for R3, and 186 patients (76%) for R4. On baseline MRI, most patients presented with heterogeneously dense or dense breasts on MRI: 187 patients (76%) for R1, 149 patients (61%) for R2, 184 patients (76%) for R3, and 183 patients (75%) for R4. On baseline mammography, most patients presented with heterogeneously dense or dense breasts: 93 patients (83%) for R1, 81 patients (72%) for R2, 89 patients (79%) for R3, and 95 patients (85%) for R4.

Fifty-one of 298 (17%) patients had a recurrence. Two of 298 were metastatic at the time of diagnosis. Of the 51 patients who had a recurrence, 13 patients had local recurrence after an average of 6.5 ± 3.3 years (1/13 had a second distant recurrence), 7 patients had a regional recurrence after 7.7 ± 3 years (4/7 patients had a second recurrence (3 distant, 1 local contralateral)), 19 patients had a distant recurrence after 7.5 ± 4.1 years, 6 patients had a local contralateral recurrence after 8.1 ± 2.1 years (1/6 had a second distant recurrence after 5 years), and 5 patients developed a contralateral malignancy of different histology after 7.2 ± 3.9 years. Twenty-nine patients died during the follow-up period: 21 deaths were related to breast cancer, 3 were for unknown reasons, and 5 were for other malignancies (non-Hodgkin’s lymphoma, pancreatic cancer, and melanoma).

### Recurrence-free survival and overall survival

**Fig. 1:**
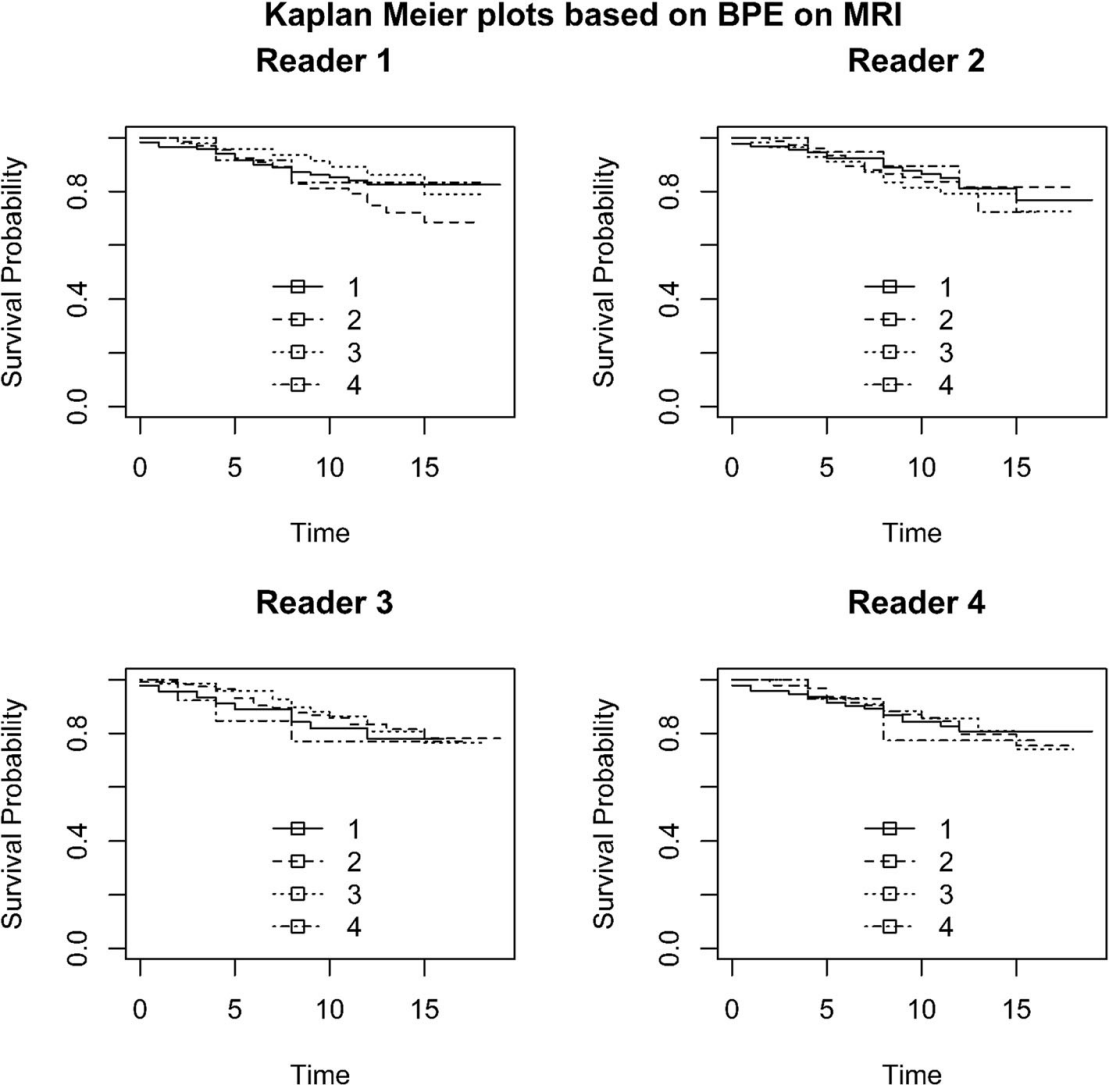
Kaplan–Meier plot of recurrence-free survival using BPE categories on MRI as strata: (1) minimal, (2) mild, (3) moderate, and (4) marked

**Fig. 2:**
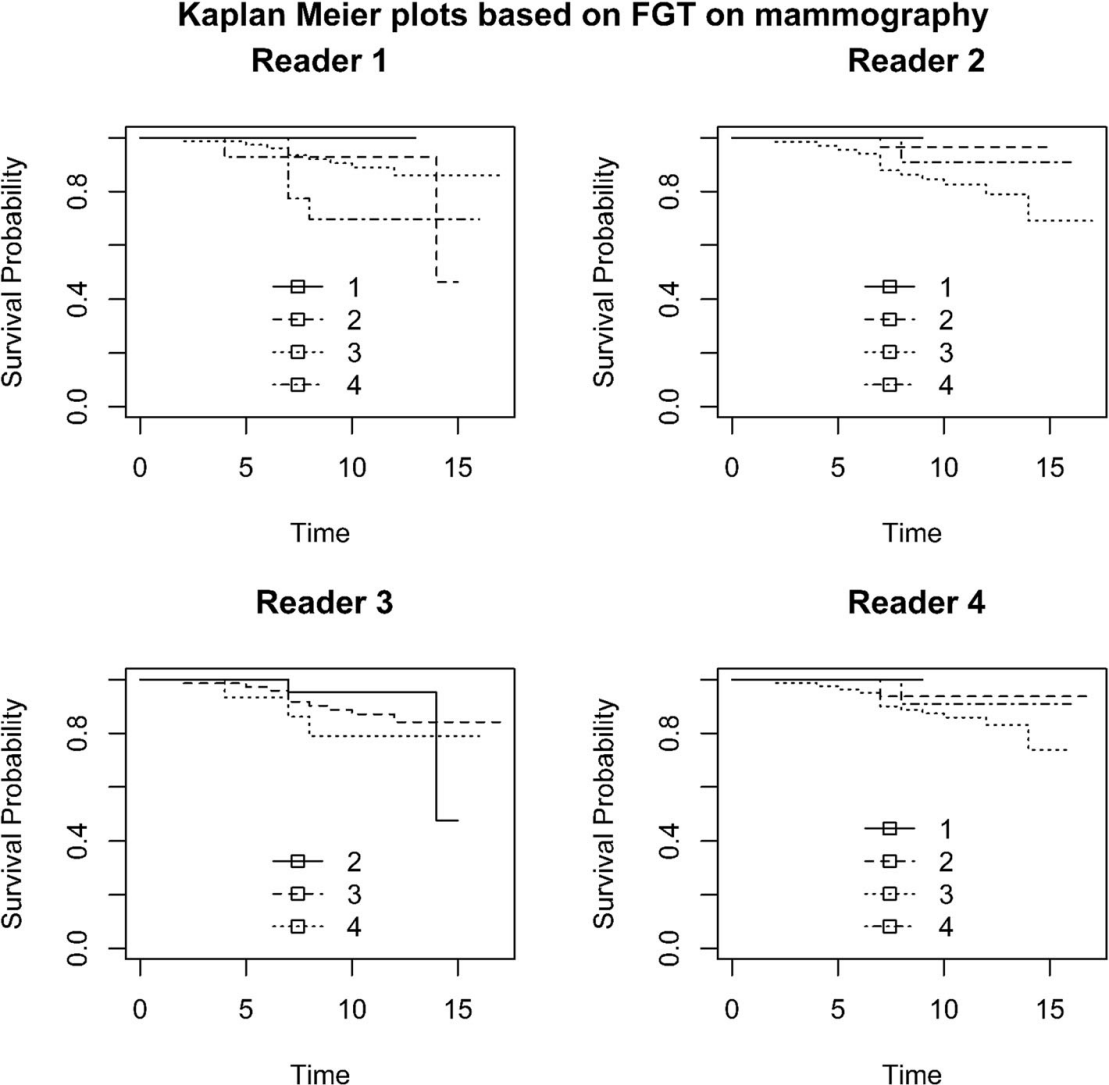
Kaplan–Meier plot of recurrence-free survival using breast density categories on mammography as strata: (1) fatty, (2) scattered fibroglandular, (3) heterogeneously dense, and (4) extremely dense

**Fig. 3:**
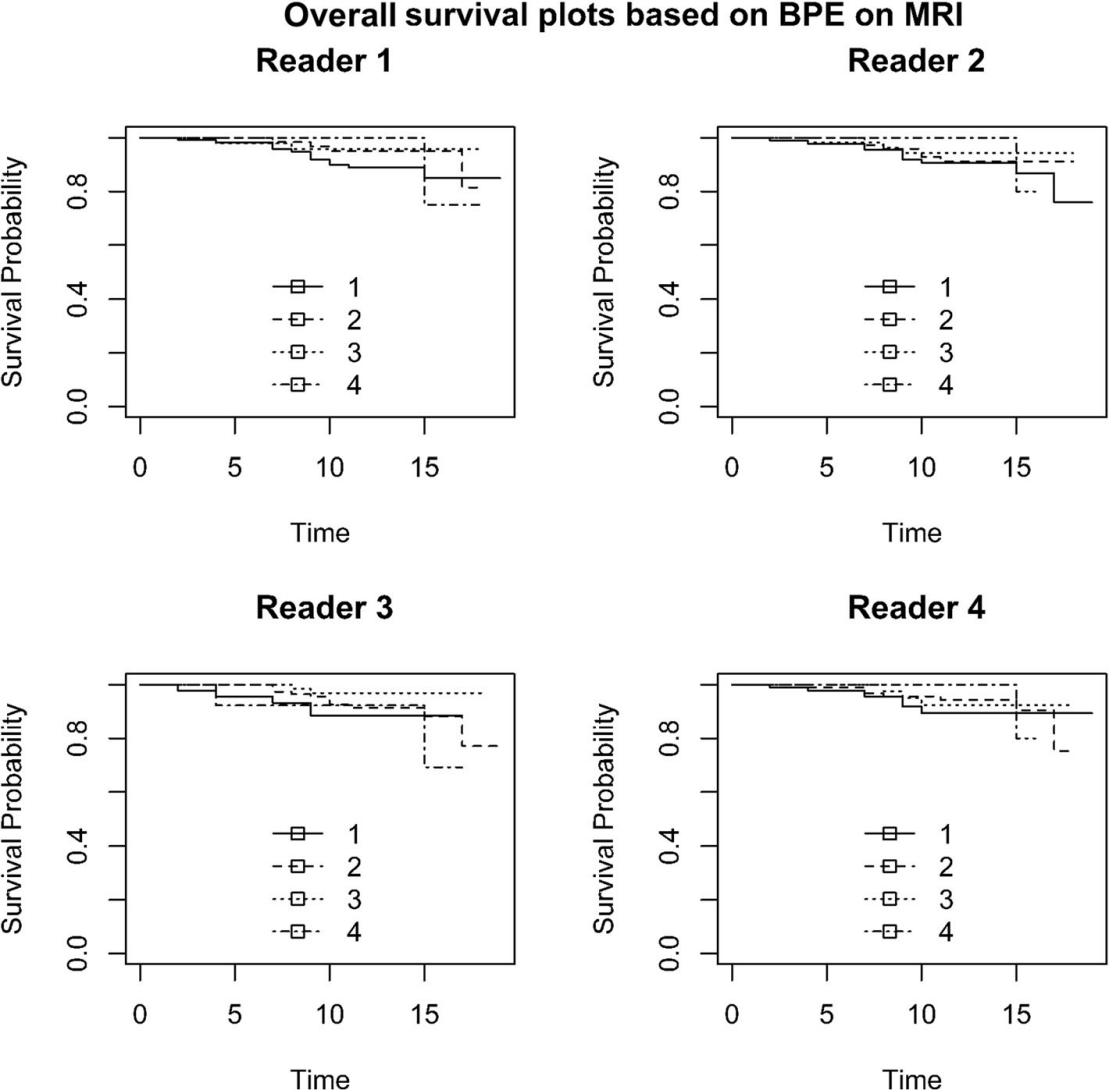
Kaplan–Meier plot of overall survival using BPE categories on MRI as strata: (1) minimal, (2) mild, (3) moderate, and (4) marked

Figures 1 and 2 and Additional File 4 (Fig. A4) show the Kaplan–Meier curves of recurrence-free survival stratified by BPE, FGT, and MD categories, respectively. The survival curves were not significantly different between BPE, FGT, and MD. Figure 3 and Additional File 5 (Fig. A5) show the Kaplan–Meier curves of overall survival stratified by BPE and FGT (the association between MD and overall survival was not measured due to paucity of data). The survival curves were not significantly different between BPE and FGT.

In order to overcome the issue of collinearity between the main covariates of interest, namely BPE, FGT, and MD, we constructed three different Cox regression models each for recurrence-free survival and overall survival using these covariates separately in each of them.

Table 3 shows the results of the Cox regression models using recurrence-free survival as the main outcome. Both the model using BPE as a covariate and the model using FGT as a covariate showed a significant association between the number of metastatic lymph nodes (*p* < 0.0001 for no metastatic lymph nodes vs. > 3 metastatic lymph nodes) and hormonal treatment duration (*p* < 0.0001 for ≥ 6 years vs. 1–5 years) with recurrence-free survival. When we re-ran the model using BPE as a covariate only in patients who had non-minimal (mild/moderate/significant) baseline BPE (excluding patients with minimal baseline BPE as none of them had moderate or marked decrease at follow-up MRI), we found a significant association between the number of metastatic lymph nodes (*p* < 0.001 for no metastatic lymph nodes vs. > 3 metastatic lymph nodes), hormonal treatment duration (*p* < 0.0001 for ≥ 6 years vs. 1–5 years), and also lesion diameter (*p* = 0.03 for micro lesions up to 19 mm vs. lesions measuring > 50 mm) with recurrence-free survival.

**Table 3:** Cox regression models using recurrence-free survival as the main outcome of interest after backward selection

	Covariate: BPE		Covariate: BPE (limited subset)		Covariate: FGT		Covariate: breast density	
	Log (HR)	*p* value	Log (HR)	*p* value	Log (HR)	*p* value	Log (HR)	*p* value
Hormonal treatment duration								
1–5 years	1.52	< .0001	1.52	< .0001	1.61	< .0001	3.17	0.02
≥ 6 years	Ref		Ref		Ref		Ref	
Number of metastatic lymph nodes								
> 3	2.49	< .0001	1.86	< 0.001	2.64	< .0001	3.11	0.001
Micro to 3	0.62	0.08	0.44	0.29	0.79	0.03	1.52	0.06
Non-metastatic	Ref		Ref		Ref		Ref	
Diameter								
20–49 mm			− 0.62	0.28				
> 50 mm			1.16	0.03				
Micro to 19 mm			Ref					

Limited subset: Only individuals with mild/moderate/significant parenchymal enhancement were included in the limited subset analysis

*Abbreviations*: *BPE* background parenchymal enhancement, *FGT* fibroglandular tissue, *HR* hazard ratio

Table 4 shows the results of the Cox regression models using recurrence-free survival (onset of recurrence local/regional or distant after a standard treatment) as the main outcome. The model using BPE as a covariate showed a significant association between age (*p* = 0.002), number of metastatic lymph nodes (*p* < 0.0001 for no metastatic lymph nodes vs. > 3 metastatic lymph nodes), hormonal treatment duration (*p* = 0.001 for ≥ 6 years vs. 1–5 years), and histology (*p* = 0.001 for classic vs. pleomorphic) with recurrence-free survival. The model using FGT as a covariate showed a significant association between age (*p* = 0.01), number of metastatic lymph nodes (*p* < 0.001 for > 3 metastatic lymph nodes vs. no metastatic lymph node), hormonal treatment duration (*p* < 0.0001 for ≥ 6 years vs. 1–5 years), and histology (*p* < 0.0001 for classic vs. pleomorphic) with recurrence-free survival. When we re-ran the model using BPE as a covariate only in patients who had non-minimal BPE, classic vs. pleomorphic histology was no longer significantly associated with recurrence-free survival.

**Table 4:** Cox regression models using recurrence-free survival as the main outcome of interest after backward selection

	Covariate: BPE		Covariate: BPE (limited subset)		Covariate: FGT	
	Log (HR)	*p* value	Log (HR)	*p* value	Log (HR)	*p* value
Age	0.06	0.002	0.06	0.05	0.07	0.01
Hormonal treatment duration						
1–5 years	2.82	0.001	2.33	0.001	3.02	< .0001
≥ 6 years	Ref		Ref		Ref	
Number of metastatic lymph nodes						
> 3	4.50	< .0001	4.12	< .0001	5.04	< .0001
Micro to 3	1.08	0.10	0.52	0.54	1.45	0.03
Non-metastatic	Ref		Ref		Ref	
Histology						
Classic	−2.02	0.001	−0.62	0.28	− 2.26	< .0001
Mixed	−2.06	0.05	1.16	0.03	− 3.29	0.06
Pleomorphic	Ref		Ref		Ref	

Limited subset: Only individuals with mild/moderate/significant parenchymal enhancement were included in the limited subset analysis

*Abbreviations*: *BPE* background parenchymal enhancement, *FGT* fibroglandular tissue, *HR* hazard ratio

### Inter-reader agreement

Table 5 shows the inter-reader agreement between the readers for assessment of BPE, change in BPE, FGT, and change in FGT. There was moderate to substantial agreement between the readers for baseline BPE (*τ* = 0.55–0.69) and moderate agreement for change in BPE (*τ* = 0.49–0.59). There was substantial inter-reader agreement (*τ* = 0.68–0.77) for baseline FGT but only fair to moderate agreement (*τ* = 0.29–0.47) for change in FGT.

**Table 5:** Inter-reader agreement between the readers for assessment of BPE, change in BPE, FGT, and change in FGT on MRI

	R1	R2	R3	R4
**R1**	*n* = 245	*n* = 243	*n* = 245	*n* = 244
BPE	1.00	0.63	0.69	0.62
Change in BPE	1.00	0.57	0.55	0.52
FGT	1.00	0.69	0.77	0.74
Change in FGT	1.00	0.47	0.40	0.42
**R2**	*n* = 243	*n* = 243	*n* = 243	*n* = 242
BPE	0.63	1.00	0.62	0.55
Change in BPE	0.57	1.00	0.59	0.51
FGT	0.69	1.00	0.69	0.68
Change in FGT	0.47	1.00	0.45	0.29
**R3**	*n* = 245	*n* = 243	*n* = 245	*n* = 244
BPE	0.69	0.62	1.00	0.62
Change in BPE	0.55	0.59	1.00	0.49
FGT	0.77	0.69	1.00	0.71
Change in FGT	0.40	0.45	1.00	0.33
**R4**	*n* = 244	*n* = 242	*n* = 244	*n* = 244
BPE	0.62	0.55	0.62	1.00
Change in BPE	0.52	0.51	0.49	1.00
FGT	0.74	0.58	0.71	1.00
Change in FGT	0.41	0.29	0.33	1.00

*n* denotes the number of observations

*Abbreviations*: *BPE* background parenchymal enhancement, *FGT* fibroglandular tissue

Table 6 shows the inter-reader agreement between the readers for the assessment of MD and change in MD. There was moderate to substantial inter-reader agreement for MD on mammography (*τ* = 0.59–0.78) but only fair to moderate agreement (*τ* = 0.33–0.58) for change in MD.

**Table 6:** Inter-reader agreement between the readers for the assessment of breast density and change in breast density on mammography

	R1 (*n* = 112)	R2 (*n* = 112)	R3 (*n* = 112)	R4 (*n* = 112)
**R1**				
Breast density	1.00	0.65	0.78	0.71
Change in breast density	1.00	0.33	0.47	0.48
**R2**				
Breast density	0.65	1.00	0.64	0.59
Change in breast density	0.33	1.00	0.42	0.40
**R3**				
Breast density	0.78	0.64	1.00	0.69
Change in breast density	0.47	0.42	1.00	0.58
**R4**				
Breast density	0.71	0.58	0.69	1.00
Change in breast density	0.48	0.40	0.58	1.00

“*n*” denotes the number of observations

There was poor agreement between BPE and FGT for all readers (*τ* = 0.19 for R1; 0.21 for R2; 0.11 for R3 and R4). Similarly, there was poor agreement between the change in BPE and the change in FGT for all readers (*τ* = 0.2 for R1, 0.11 for R2, 0.18 for R3, and 0.21 for R4).

## Discussion

Among patients with breast cancer, patients with ILC receive the most benefit from endocrine treatment [11]. To the best of our knowledge, no study has evaluated the association between BPE, FGT, and MD and the risk of distant metastasis or breast cancer-related mortality in patients with ILC undergoing adjuvant treatment. Our study demonstrated that neither BPE, FGT, and MD at baseline nor change in BPE, FGT, or MD from the pre-treatment and first post-endocrine treatment imaging was significantly associated with overall and recurrence-free survival. The duration of endocrine therapy showed a significant association with recurrence and recurrence-free survival. Other variables that seemed to have an impact were the number of metastatic axillary lymph nodes, histology, patient age, and tumor diameter.

The risk of breast cancer has been shown to increase steadily with increasing MD [27]. Studies have shown that MD correlates with FGT on MRI [28–30], and it has been suggested that since breast MRI is three-dimensional, it may enable a more accurate assessment [31–35]. In our study, there was a significant correlation between FGT and MD for all for readers (*p* < 0.001). The association between mammographic MD and BPE remains controversial. Two prior studies [36, 37] found no correlation between BPE and mammographic density; however, other studies have shown that BPE is directly correlated with mammographic density [38–40]. In our study, there was no correlation between BPE and MD (*p* = 0.43 for R1, *p* = 0.61 for R2, *p* = 0.38 for R3, and *p* = 0.4 for R4).

Several studies have demonstrated that BPE is associated with breast cancer risk independent of breast density. King et al. [17] demonstrated that the odds ratio of breast cancer in high-risk patients significantly increased with high BPE. This was confirmed by another study published by Dontchos et al. [18]. However, a study by Bennani-Baiti et al. [41] in a non-high-risk population (estimated lifetime risk < 20%) found no association between BPE and FGT and breast cancer risk in multivariate analysis (odds ratio 1.249; 95% confidence interval 0.469–3.332). Previous studies have suggested that BPE and to a lesser extent FGT are impacted by endocrine treatment such as aromatase inhibitors [42] and tamoxifen [43–45]. While the fact that BPE may represent a risk factor for breast cancer and that they are both reduced by endocrine treatment could signify that they may be used as a biomarker of treatment response, our results do not support their use as imaging biomarkers—at least in patients with ILC.

As of today, conflicting results have been published about the prognostic impact of BPE at the time of breast cancer diagnosis. In a study by Van der Velden at al [46]. that included 302 patients with IDC whose BPE was assessed automatically, patients with high baseline contralateral BPE had significantly better survival compared to patients with low BPE. In another study by Van der Velden et al. [19], in which 75/531 (14%) of the study cohort had ILC, patients with lower enhancement values had a less favorable therapy outcome than those with higher enhancement values. Contrary to Van der Velden, a study by Lim et al. [22] that included 804 women with invasive breast cancer demonstrated that increased pre-operative BPE in post-menopausal women was predictive of poor recurrence-free survival; however, there was no correlation between BPE and patient outcome in pre-menopausal women. This study by Lim et al. included both IDC, ILC, and other breast cancers. There were only 22 ILCs (2.7%), and only one patient with ILC had a recurrence. Similar to our results, a study by Shin et al. [47] in 289 patients with ER+, HER2−, node-negative invasive breast cancer showed no correlation between contralateral BPE and survival outcome. A study by Kim et al. [48] showed that increased BPE was a predictor of a poor breast cancer prognosis (larger diameter and EGFR positivity).

In patients undergoing neoadjuvant chemotherapy, a study by Preibsch et al. [20] demonstrated that the degree of BPE reduction (visually assessed by two radiologists) seemed to correlate with tumor response. This study was performed in 73 patients with 80 cancers and 10% of which were ILCs. It did not evaluate response to adjuvant endocrine treatment. A similar study by Dong et al. [49] produced similar results in HER2+ tumors. In patients undergoing reducing salpingo-oophorectomy, Bermot et al. [50] showed that the reduction in BPE and FGT before and after risk-reducing salpingo-oophorectomy in women with high genetic risk of breast cancer was correlated with a higher risk of subsequent breast cancer compared to patients with stable BPE. This is in contrast with a study by DeLeo et al. [51] that showed that mean BPE after risk-reducing salpingo-oophorectomy remained higher in women with subsequent cancer than in patients without cancers.

The correlation between breast fibroglandular tissue and prediction of survival has not been investigated. King et al. [17] assessed breast cancer risk and its association with FGT, yet this was not as strong as with BPE. Donchos et al. [18] reported no significant association between amounts of FGT or MD and cancer development (*p* = 0.5 and *p* = 0.4, respectively). This is similar to our results which show that neither FGT nor MD is useful imaging biomarkers to assess survival.

Our study has several limitations. First, both BPE and FGT were qualitatively graded. This subjective method of assessment is an inherent limitation. However, it accurately reflects the current clinical practice and we further tried to mitigate this limitation by using four experienced readers. Especially for the evaluation of changes in the amount of BPE and FGT, the inter-reader agreement was fair to moderate and could potentially have masked associations. Automated quantitative rather than qualitative evaluation may potentially help reduce this variability and should be the topic of future studies. Second, the retrospective nature of this study did not allow us to analyze the point of the menstrual cycle at each MRI examination; further subgroup analysis based on the time of the menstrual cycle could strengthen the results of this study. Third, as previously observed in studies involving patients undergoing neoadjuvant therapy, chemotherapy influences MD and BPE [52]. In our study, 193/298 patients underwent chemotherapy. Of these, 171 patients began chemotherapy after baseline MRI and mammography. The remaining 22 patients underwent baseline MRI after the first cycle of chemotherapy; 7/22 patients were evaluated as having no changes in BPE and FGT by all readers after the onset of hormonal treatment. It has to be noted that it was not possible to stratify by the menopausal status as this information was not available in every patient. Therefore, additional studies with a larger sample size and with adjustment for other risk factors (menstrual, menopausal status, and familial risks of breast cancer, age, BRCA mutation) are needed to evaluate whether there is an association between BPE, FGT, and MD with patient survival.

## Conclusions

In conclusion, qualitative evaluation of BPE, FGT, and mammographic MD changes cannot predict which patients are more likely to benefit from adjuvant endocrine treatment. Adjuvant endocrine treatment duration is associated with survival in women diagnosed with ILC.

## Supplementary information

**Additional file 1: Fig. A1.** Contrast-enhanced T1-weighted fat-suppressed subtraction maximum intensity projection images in the sagittal plane showing examples of minimal, mild, moderate and marked BPE, respectively.

**Additional file 2: Fig. A2.** Contrast-enhanced T1-weighted fat-suppressed images (A,B) and corresponding subtraction maximum intensity projection images (C,D) in the sagittal plane before (A,C) and after (B,D) onset of endocrine treatment showing mild change in BPE.

**Additional file 3: Fig. A3.** Contrast-enhanced T1-weighted fat-suppressed images (A,B) and corresponding subtraction maximum intensity projection images (C,D) in the sagittal plane before (A,C) and after (B,D) onset of endocrine treatment showing marked change in BPE.

**Additional file 4: Fig. A4.** Kaplan–Meier plot of recurrence-free survival using FGT categories on MRI as strata. 1) almost entirely fat; 2) scattered fibroglandular tissue; 3) heterogeneous fibroglandular tissue; 4) extreme fibroglandular tissue.

**Additional file 5: Fig. A5.** Kaplan–Meier plot of overall survival using FGT categories on MRI as strata. 1) almost entirely fat; 2) scattered fibroglandular tissue; 3) heterogeneous fibroglandular tissue; 4) extreme fibroglandular tissue.

## Data Availability

The datasets used and/or analyzed during the current study are available from the corresponding author on reasonable request.

## Abbreviations

BI-RADS: Breast Imaging Reporting and Data System
BPE: Background parenchymal enhancement
FGT: Fibroglandular tissue
IDC: Invasive ductal carcinoma
ILC: Invasive lobular carcinoma
MD: Mammographic density
MRI: Magnetic resonance imaging
