# Supplementary material for: MRI background parenchymal enhancement, fibroglandular tissue, and mammographic breast density in patients with invasive lobular breast cancer on adjuvant endocrine hormonal treatment: associations with survival
Source: Breast Cancer Res. 2020 Aug 20;22:93. doi: 10.1186/s13058-020-01329-z (PMC7441557; doi:10.1186/s13058-020-01329-z)

Additional File 1

Figure A1: Contrast-enhanced T1-weighted fat-suppressed subtraction maximum intensity projection images in the sagittal plane showing examples of minimal, mild, moderate and marked BPE, respectively.

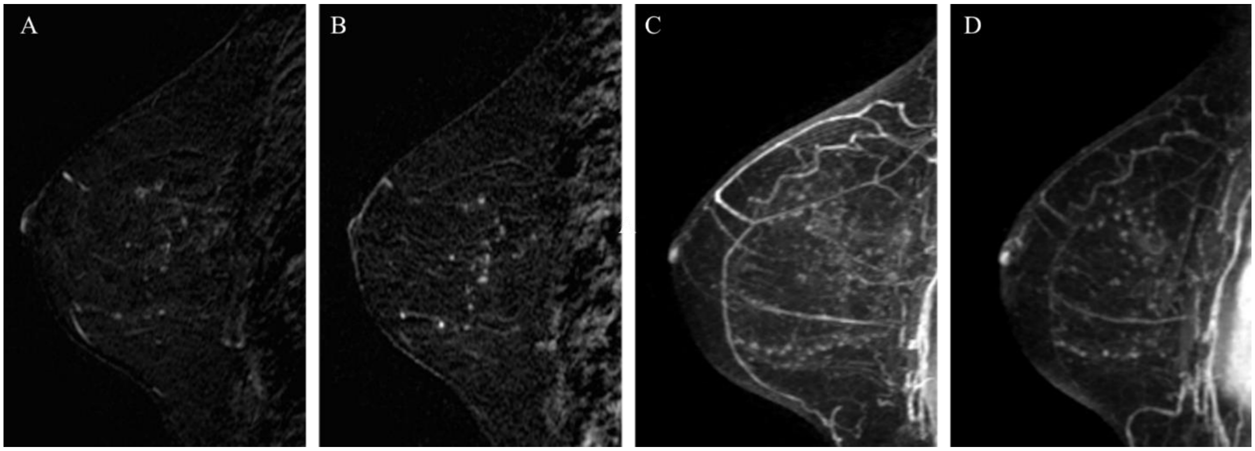

Supplement: Supplementary file 1 — Additional file 1: Fig. A1. Contrast-enhanced T1-weighted fat-suppressed subtraction maximum intensity projection images in the sagittal plane showing examples of minimal, mild, moderate and marked BPE, respectively. [file 13058_2020_1329_MOESM1_ESM.pdf]
