# Supplementary material for: MRI background parenchymal enhancement, fibroglandular tissue, and mammographic breast density in patients with invasive lobular breast cancer on adjuvant endocrine hormonal treatment: associations with survival
Source: Breast Cancer Res. 2020 Aug 20;22:93. doi: 10.1186/s13058-020-01329-z (PMC7441557; doi:10.1186/s13058-020-01329-z)

Additional File 2

Figure A2: Contrast-enhanced T1-weighted fat-suppressed images (A,B) and corresponding subtraction maximum intensity projection images (C,D) in the sagittal plane before (A,C) and after (B,D) onset of endocrine treatment showing mild change in BPE.

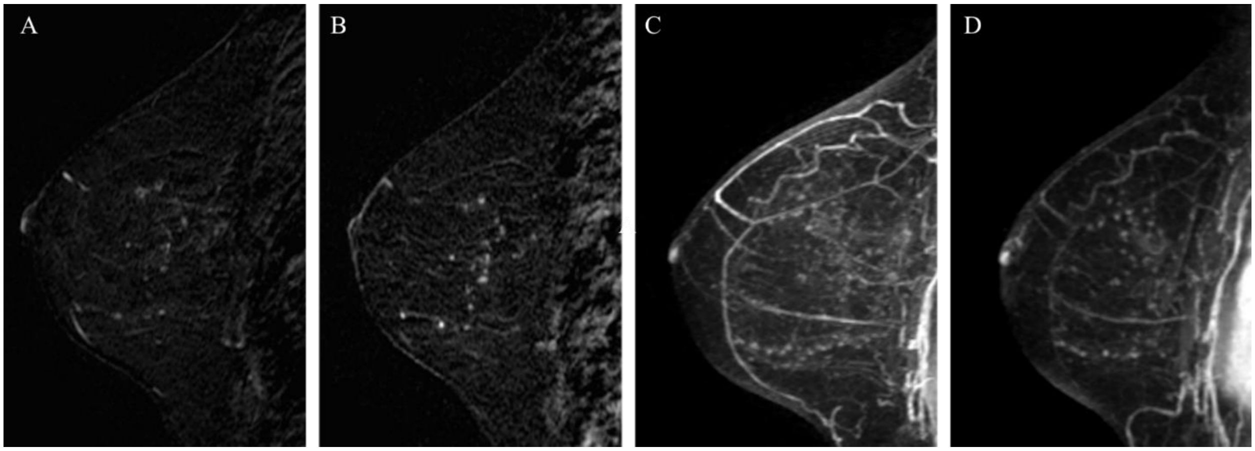

Supplement: Supplementary file 2 — Additional file 2: Fig. A2. Contrast-enhanced T1-weighted fat-suppressed images (A,B) and corresponding subtraction maximum intensity projection images (C,D) in the sagittal plane before (A,C) and after (B,D) onset of endocrine treatment showing mild change in BPE. [file 13058_2020_1329_MOESM2_ESM.pdf]
