# Supplementary material for: MRI background parenchymal enhancement, fibroglandular tissue, and mammographic breast density in patients with invasive lobular breast cancer on adjuvant endocrine hormonal treatment: associations with survival
Source: Breast Cancer Res. 2020 Aug 20;22:93. doi: 10.1186/s13058-020-01329-z (PMC7441557; doi:10.1186/s13058-020-01329-z)

Additional File 4

Figure A4: Kaplan–Meier plot of recurrence-free survival using FGT categories on MRI as strata. 1) almost entirely fat; 2) scattered fibroglandular tissue; 3) heterogeneous fibroglandular tissue; 4) extreme fibroglandular tissue.

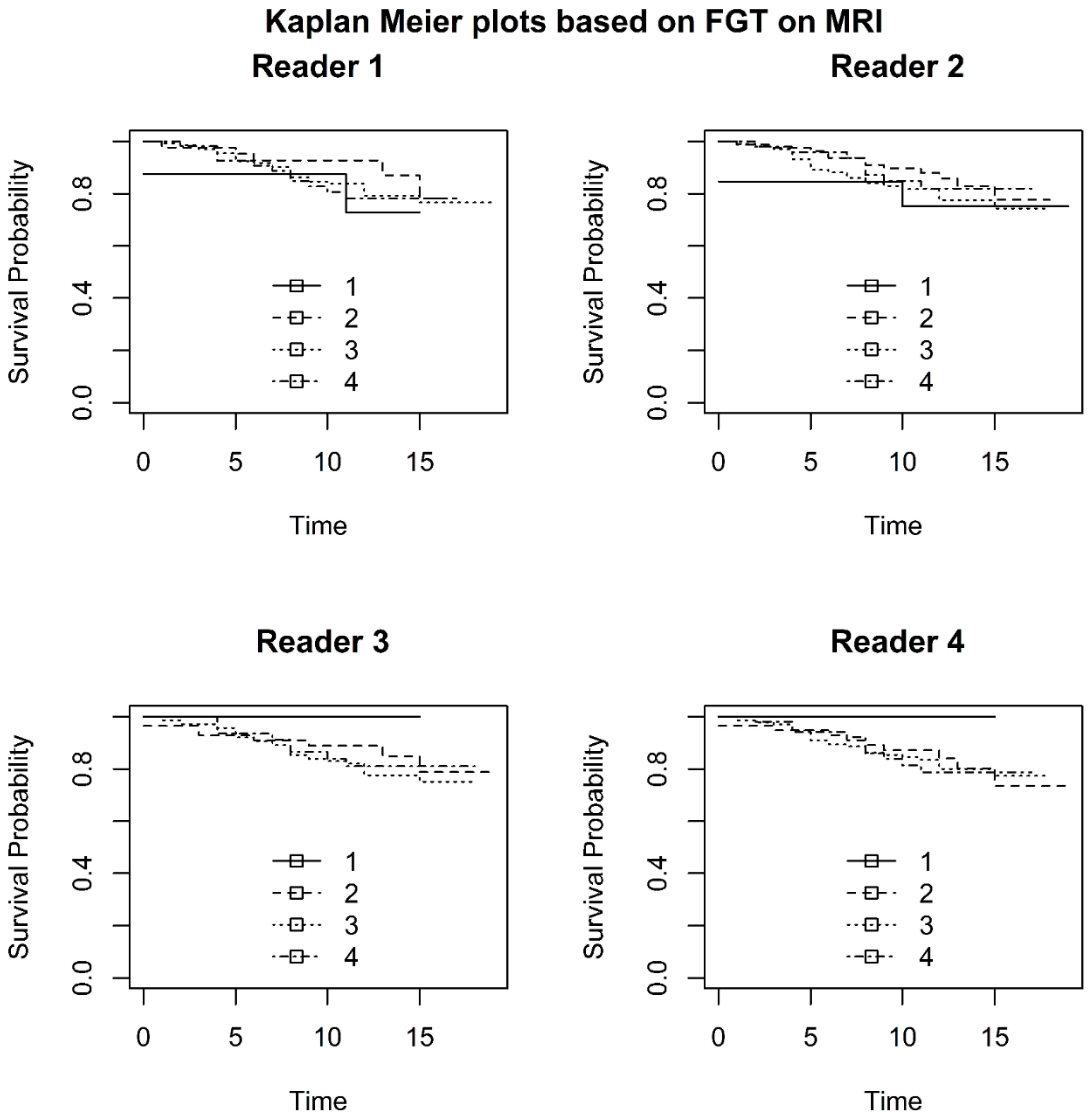

Supplement: Supplementary file 4 — Additional file 4: Fig. A4. Kaplan–Meier plot of recurrence-free survival using FGT categories on MRI as strata. 1) almost entirely fat; 2) scattered fibroglandular tissue; 3) heterogeneous fibroglandular tissue; 4) extreme fibroglandular tissue. [file 13058_2020_1329_MOESM4_ESM.pdf]
